# Evaluating the Histopathology of Pancreatic Ductal Adenocarcinoma by Intravoxel Incoherent Motion-Diffusion Weighted Imaging Comparing With Diffusion-Weighted Imaging

**DOI:** 10.3389/fonc.2021.670085

**Published:** 2021-06-23

**Authors:** Qi Liu, Jinggang Zhang, Man Jiang, Yue Zhang, Tongbing Chen, Jilei Zhang, Bei Li, Jie Chen, Wei Xing

**Affiliations:** ^1^ Department of Radiology, The Third Affiliated Hospital of Soochow University, Changzhou, China; ^2^ Department of Hepatobiliary and Pancreatic Surgery, The Third Affiliated Hospital of Soochow University, Changzhou, China; ^3^ Department of Pathology, The Third Affiliated Hospital of Soochow University, Changzhou, China; ^4^ Clinical Science, Philips Healthcare, Shanghai, China

**Keywords:** pancreatic ductal adenocarcinoma, diffusion-weighted imaging, intravoxel incoherent motion-diffusion weighted imaging, fibrosis, biomarker

## Abstract

**Objectives:**

To explore the differences between intravoxel incoherent motion diffusion-weighted imaging (IVIM-DWI) and diffusion-weighted imaging (DWI) in evaluating the histopathological characters of pancreatic ductal adenocarcinoma (PDAC).

**Methods:**

This retrospective study enrolled 50 patients with PDAC confirmed by pathology from December 2018 to May 2020. All patients underwent DWI and IVIM-DWI before surgeries. Patients were classified into low- and high-fibrosis groups. Apparent diffusion coefficient (ADC), diffusion coefficient (D), false diffusion coefficient (D*), and perfusion fraction (f) were measured by two radiologists, respectively in GE AW 4.7 post-processing station, wherein ADC values were derived by mono-exponential fits and f, D, D* values were derived by biexponential fits. The tumor tissue was stained with Sirius red, CD34, and CK19 to evaluate fibrosis, microvascular density (MVD), and tumor cell density. Furthermore, the correlation between ADC, D, D*, and f values and histopathological results was analyzed.

**Results:**

The D values were lower in the high-fibrosis group than in the low-fibrosis group, while the f values were opposite. Further, no statistically significant differences were detected in ADC and D* values between the high- and low-fibrosis groups. The AUC of D and f values had higher evaluation efficacy in the high- and low-fibrosis groups than ADC values. A significant negative correlation was established between D values, and fibrosis and a significant positive correlation were observed between f values and fibrosis. No statistical difference was detected between DWI/IVIM parameters values and MVD or tumor cell density except for the positive correlation between D* values and tumor cell density.

**Conclusions:**

D and f values derived from the IVIM model had higher sensitivity and diagnostic performance for grading fibrosis in PDAC compared to the conventional DWI model. IVIM-DWI may have the potential as an imaging biomarker for predicting the fibrosis grade of PDAC.

## Introduction

Pancreatic ductal adenocarcinoma (PDAC) is one of the most malignant cancers with a 5-year survival rate <10% in the USA (1). Complete surgical resection and chemotherapy could provide the highest survival time, but 80% of patients are unresectable. Systemic chemotherapy is the most important and basic treatment of PDAC, which could relieve cancer symptoms and prolong life. In addition to prolonging survival and relieving symptoms, 10–30% of patients shift from unresectable pancreatic cancer to resectable pancreatic cancer after chemotherapy (2).

However, the result of chemotherapy on PDAC patients is inconsistent from no reaction to complete remission. Tumor microenvironment (TME) directly affects the effect of chemotherapy in PDAC. The TME consists of acellular stroma, immune cells, pancreatic stellate cells, and soluble factors (3). The fibrosis creates a mechanical barrier wrapping around the tumor, limiting vascularization, hindering access to chemotherapy, and limiting immune cell infiltration (4). Thus, new therapies that target tumor cells and fibrous tissue are required (5–7). The monitoring of therapy and treatment planning would benefit from assessing the fibrosis non-invasively and correctly.

Diffusion-weighted imaging (DWI) measures the ADC values using a single exponential model, which could quantitatively reflect the diffusion motion of water molecules in tissues (8). DWI detects the physiological characteristics of tissue non-invasively by measuring the diffusion properties of water molecules (9). Several studies have applied the mean ADC value, which was significantly higher in the loose fibrosis group PDAC than in the dense fibrosis group and negatively correlated with PDAC fibrosis (10–12). Also, Klauss et al. did not find any statistical difference in the ADC values between high- and low-fibrosis PDAC (13).

However, the ADC values obtained from the traditional DWI contain both water molecule diffusion and microcirculatory perfusion. As an advanced magnetic resonance (MR) sequence, IVIM was first shown by Le Bihan et al. (14). IVIM-DWI with the derived parameters of pure molecular diffusion coefficient (D), perfusion fraction (f), and perfusion-related diffusion coefficient (D*) can separate water molecule diffusion and microcirculatory perfusion-related diffusion, thereby compensating for the shortcomings of traditional DWI. Several studies had applied IVIM-DWI for better diagnostic performance than traditional ADC values when breast cancer was discriminated from benign breast lesions, or the histological subtypes of breast cancer were characterized (15–17). Ma et al. showed D_slow_ and f values as predictors of PDAC grades (including fibrosis), and both cases with an area under the curve (AUC) >0.85, which proved that IVIM was indeed an advanced MR technology (18). However, only a few previous studies about IVIM assessed fibrosis in pancreatic cancer, and the methods and results are inconsistent (11, 13, 19). Hitherto, whether the IVIM-DWI techniques in assessing the degree of fibrosis in PDAC is superior to DWI techniques has not yet been studied. This study compares the efficacy of DWI and IVIM-DWI in assessing PDAC fibrosis by assessing the association between imaging and pathological parameters.

## Materials And Methods

This study was conducted in accordance with the Helsinki Declaration and approved by the Third Affiliated Hospital of Soochow University, Institutional Ethics Committee, and written informed consent for the study participants was waived by the ethics committee.

### Patients

**Figure 1:**
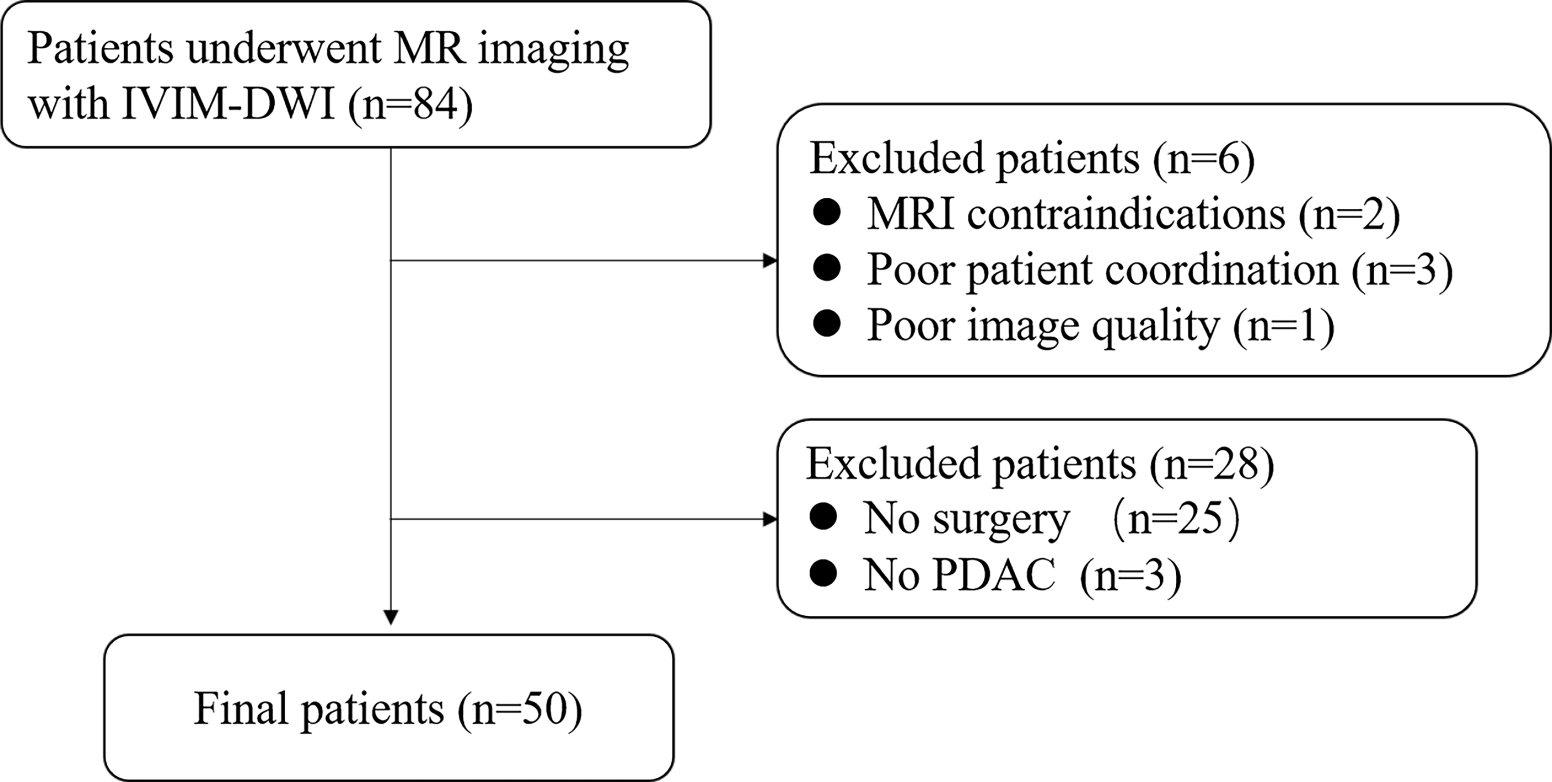
Schematic of the patient selection process.

From November 2018 to May 2020, a total of 73 consecutive patients diagnosed with PDAC by computed tomography (CT) were analyzed in this retrospective study. The inclusion criteria were as follows: (1) complete routine pancreatic MRI, DWI, and IVIM-DWI within 1 week before surgery; (2) no previous treatment before surgery; (3) postoperative pathology confirmed PDAC. The exclusion criteria were as follows: MRI contraindications, poor patient coordination, or poor image quality. Finally, 50 non-consecutive patients were included in the current study, and the detailed flowchart is shown in **Figure 1**.

### Imaging Technique

The data were acquired on a 3.0 Tesla MRI system (Ingenia, Philips Healthcare, Best, Netherlands) using a standard 32-channel phased-array coil. All patients were on an empty stomach for 6–8 h before MR examination and underwent breath-holding training. MR scanning was performed with respiratory gating. The detailed MRI protocol is described in **Table 1**. Axial DWI sequence included two b values (b = 0 and 800 s/mm^2^), and axial IVIM-DWI sequence included 10 b-values (b = 0, 10, 20, 40, 60, 80, 100, 150, 200, and 500 s/mm^2^).

**Table 1:** Magnetic resonance imaging parameters.

Parameters	Sequences				
	Coronal T2WI	Axial T1WI	Axial T2WI	Axial DWI	Axial IVIM-DWI
Repetition time, ms	900	4	2,300	400	1,261
Echo time, ms	80	0	80	63	69
Field of view, mm^2^	350 × 382	380 × 317	380 × 380	120 × 120	380 × 298
Matrix size, mm^2^	220 × 209	280 × 208	280 × 280	64 × 63	128 × 98
Slice thickness, mm	5.0	4.5	6.5	4.0	4.0
Slice gap, mm	1.0	–2.25	1.0	0.4	1.0
Flip angle, °	90	10	90	90	90
Bandwidth, Hz/pixel	127.5	362.3	357.1	1.4	4.6
Acquisition Time, s	21	12	135	316	414

### Imaging Analysis

All MR images were analyzed on an AW 4.7 post-processing workstation (Discovery silent, GE Healthcare, USA) using the Functool-MADC software. The ADC value was calculated using a mono-exponential model according to the equation:

$$\text{S}/{\text{S}}_{0}=\text{exp}(-\text{b}\cdot \text{ADC})$$

The IVIM-DWI parameters were generated using a bi-exponential model according to the equation:

$$\text{S}/{\text{S}}_{0}=(1-\text{f})\cdot \text{exp}(-\text{b}\cdot \text{D})+\text{f}\cdot \text{exp}(-\text{b}\cdot \text{D}*)$$

Where S is the signal intensity for a selected b-value, and S_0_ is the signal intensity for b = 0s/mm^2^. B-value is the diffusion sensitivity coefficient. In order to avoid the mathematical instability when the three IVIM-DWI parameters are simultaneously fitted, we used a segment analysis method described previously. Considering that blood flow perfusion is negligible in high b values, the D values are obtained using a mono-exponential model with b values > 150 mm^2^/s (20). Then, the D* and f values were fitted using the bi-exponential model when b < 150 mm^2^/s was defined as a low b-value.

**Figure 2:**
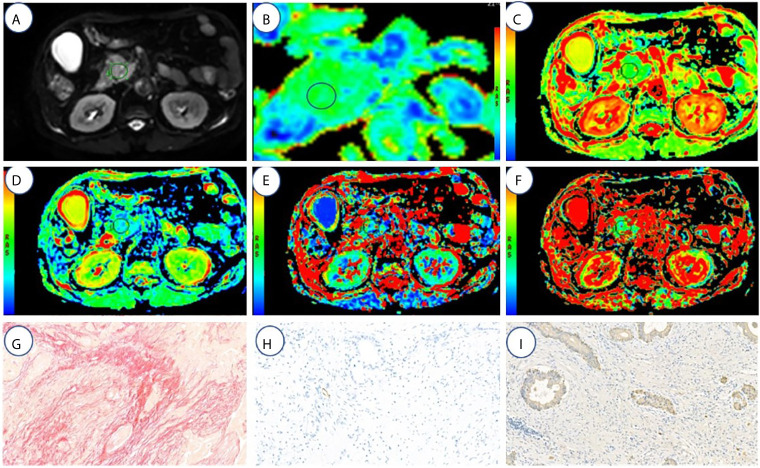
A 67-year-old male with high fibrosis PDAC. **(A–F)** represents the DWI, ADC(0,500), ADC(0,800), D, f, and D* values map, respectively. **(G–I)** represents the fibrosis, microvascular density, and tumor cell dyeing map, respectively. ROI was set in PDAC on DWI, ADC _(0, 500)_, ADC _(0, 800)_, D, f, and D* value maps. The cancer tissues show different signals from normal tissues in the DWI, ADC _(0,500)_ (1.3 μm^2^/ms), ADC _(0,800)_ (1.2 μm^2^/ms), D (1.16 μm^2^/ms), f (18.03%), and D* (64.5 μm^2^/ms). Fibrosis, MVD, and tumor cell are 47, 1.3, and 21%, respectively.

**Figure 3:**
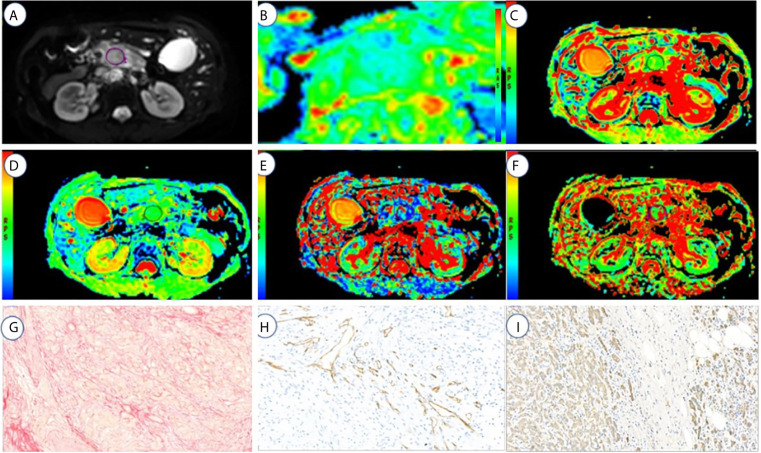
A 72-year-old male with high fibrosis PDAC. **(A–F)** represents the DWI, ADC(0,500), ADC(0,800), D, f, and D* values map, respectively. **(G–I)** represents the fibrosis, microvascular density, and tumor cell dyeing map, respectively. ROI was set in PDAC on DWI, ADC _(0, 500)_, ADC _(0, 800)_, D, f, and D* values maps. The cancer tissue showed different signals from normal tissues in the DWI, ADC _(0,500)_ (1.7 μm^2^/ms), ADC _(0,800)_ (1.6 μm^2^/ms), D (1.53 μm^2^/ms), f (13.05%), and D* (125.3 μm^2^/ms). Fibrosis, MVD, and tumor cell are 21, 6.5, and 29%, respectively.

All imaging data analysis was performed by two radiologists with 10 (JGZ) and 14 (JC) years of experience in abdominal MRI. Referring to T2-weighted and contrast-enhanced MRI data, the regions of interest (ROIs) were selected at axial maximum tumor level (**Figures 2**, **3**). Each radiologist drew two ROIs in each area of interest. The areas of ROIs were between 65.9 mm^2^ and 240.3 mm^2^ (mean, 127.2 mm^2^; median, 105 mm^2^), and the pixel counts of ROIs were between 37 and 135 (1 pixel: 1.78 mm^2^). The ROIs were performed approximately 2–3 mm from the margins of the tumor because the tumor borders were unclear. The blood vessels, catheters, necrotic areas, and calcification were excluded while drawing the ROIs. The calcification showed a low signal on T1WI and T2WI. The pancreatic duct showed a low strip signal on T1WI and a high signal on T2WI without enhancement. The blood vessels showed a low strip signal on T1WI and a low signal on T2WI with strip enhancement. The necrotic area showed a low signal on T1WI and a high signal on T2WI with no enhancement.

### Quantitative Histopathology

In order to match with axial MR images, specimens were cut into 4-mm transverse sections. Then, 5-µm-thick sections were cut from paraffin block stained with Sirius red and hematoxylin and eosin to quantitate the intratumoral fibrosis. The microvessel density (MVD) and tumor cell density were measured by CD34 and cytokeratin 19 (CK19) immunostaining of tumor tissue in an automated stainer.

A pathologist with 15 years of experience blinded to the MRI results performed the histopathology analysis. The MVD, intratumoral fibrosis, and tumor cell density were analyzed using the Image J software (version 1.47, National Institutes of Health, Bethesda, MD, USA) and five high-power fields (×200) were selected for random analysis. The percentage of fibrosis in a high-power field was defined as the ratio of the area of fibrosis tissue to that of the visual field.

According to the proportion of fibrosis in pathological features, the degree of fibrosis of PDAC was divided into four grades: Grade 1, 0–15%; Grade 2, 15–30%; Grade 3, 30–45%; Grade 4, 45–60%. Then, patients would be classified into two groups: low-fibrosis group (Grade 1 and 2); high-fibrosis group (Grade 3 and 4) (10). The fibrosis, MVD, and tumor cell density were calculated by averaging the values when the minimum and maximum values were excluded.

### Statistical Analysis

Statistical analysis was performed using SPSS (version 19.0; SPSS, Chicago, IL, USA). Intraobserver and interobserver reliability for DWI and IVIM parameters was assessed using the intraclass correlation coefficient (ICC). The ICC values were graded as follows: 0.7–1.0, strong; 0.5–0.7, moderate; 0–0.5, weak. The normality of data distribution was tested by the Kolmogorov–Smirnov test. Quantitative data with normal distribution were presented as means ± standard deviation. ADC with b _(0, 500)_, ADC with b _(0, 800)_, D, D*, and f values between high- and low-fibrosis groups of PDAC were compared by an independent sample t-test. The correlation analysis between the quantitative DWI parameters and histopathology features (fibrosis, MVD, and tumor cell density) was performed using Pearson’s correlation when the data followed a normal distribution; otherwise, Spearman’s correlation was used. The r values were assessed as follows: 0.8–1.0, strong; 0.5–0.8, moderate; 0–0.5, weak. The receiver operating characteristic (ROC) curves were plotted to assess the diagnostic performance of the ADC _(0, 500)_, ADC _(0, 800)_, D, D*, and f values in differentiating high-fibrosis from low-fibrosis PDAC. Also, accuracy, specificity, sensitivity, negative predictive value (NPV), positive predictive value (PPV), and area under the ROC curve (AUC) were calculated for each parameter. P < 0.05 indicated statistical significance.

## Results

### Repeatability

The interreader agreement in ADC _(0, 500)_, ADC _(0, 800)_, D, and f values between two observers was strong, with the ICCs ranging from 0.75 to 0.83. However, the agreement for D* values between the two observers was weak (0.23).

The intrareader agreement was strong for both observers with respect to ADC _(0, 500)_, ADC _(0, 800)_, D, f, and D* values. For observer 1, the ICCs of the ADC _(0, 500)_, ADC _(0, 800)_, f, D, and D* value ranged from 0.75 to 0.90, and for observer 2, the ICC ranged from 0.72 to 0.95.

### Comparison of DWI/IVIM Parameters Between High- and Low-Fibrosis PDAC

The ADC _(0, 500)_, ADC _(0, 800)_, f, D, and D* values of low- and high-fibrosis PDAC were 1.49 ± 0.22 *vs.* 1.43 ± 0.23 μm^2^/ms, 1.23 ± 0.11 *vs.* 1.34 ± 0.34 μm^2^/ms, 13.03 ± 6.22% *vs.* 18.80 ± 4.98%, 1.36 ± 0.18 *vs.* 1.26 ± 0.16 μm^2^/ms, and 123.05 ± 72.09 *vs.* 103.27 ± 54.23 μm^2^/ms, respectively (**Table 2**).

**Table 2:** Comparison of DWI/IVIM Parameters Between High- and Low-Fibrosis PDAC.

	High-fibrosis PDAC (n = 39)	Low-fibrosis PDAC (n = 11)	T value	P value
ADC _(0,500)_, μm^2^/ms	1.43 ± 0.23	1.49 ± 0.22	−0.76	0.45
ADC _(0,800)_, μm^2^/ms	1.34 ± 0.34	1.23 ± 0.11	−0.54	0.59
D, μm^2^/ms	1.26 ± 0.16	1.36 ± 0.18	2.25	0.03
F, %	18.80 ± 4.98	13.03 ± 6.22	−2.11	0.04
D*, μm^2^/ms	103.27 ± 54.23	123.05 ± 72.09	1.63	0.11

*P < 0.05.

The D values of the high-fibrosis group were lower than those of the low-fibrosis group (t = 2.25, P < 0.05), while the f values of the high-fibrosis group were higher than those of PDAC of the low-fibrosis group (t = −2.11, P < 0.05).

**Figure 4:**
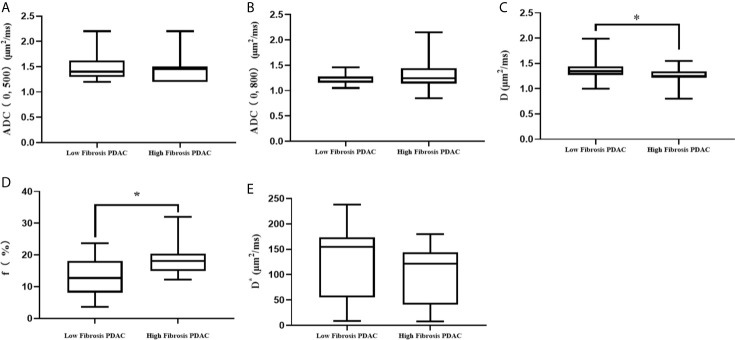
Comparison of DWI parameters between high- and low-fibrosis PDAC. **(A)** The comparison of ADC _(0, 500)_ values between low- and high-fibrosis PDAC. **(B)** The comparison of ADC _(0, 800)_ values between low- and high-fibrosis PDAC. **(C)** The comparison of D values between low- and high-fibrosis PDAC. **(D)** The comparison of f values between low- and high-fibrosis PDAC. **(E)** The comparison of D* values between low- and high-fibrosis PDAC. *P < 0.05.

No statistical differences were found in the ADC _(0, 500)_, ADC _(0, 800)_, and D* values between high-and low-fibrosis PDAC (**Figure 4**).

### Correlation Between DWI/IVIM Parameters and Histopathology Features

**Figure 5:**
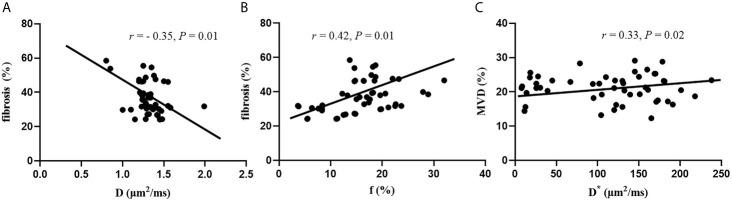
Correlation between DWI parameters and histopathology features. **(A)** Correlation between D values and fibrosis. **(B)** Correlation between f values and fibrosis. **(C)** Correlation between D* and MVD.

A significant negative correlation was established between D values and fibrosis (r = −0.35, P = 0.01). Significant positive correlations were observed between f values and fibrosis (r = 0.42, P = 0.01), and between D* values and MVD (r = 0.33, P = 0.02). No significant correlation was established between DWI parameters and pathological tissue (P > 0.05) (**Figure 5**).

### ROC Curve

**Figure 6:**
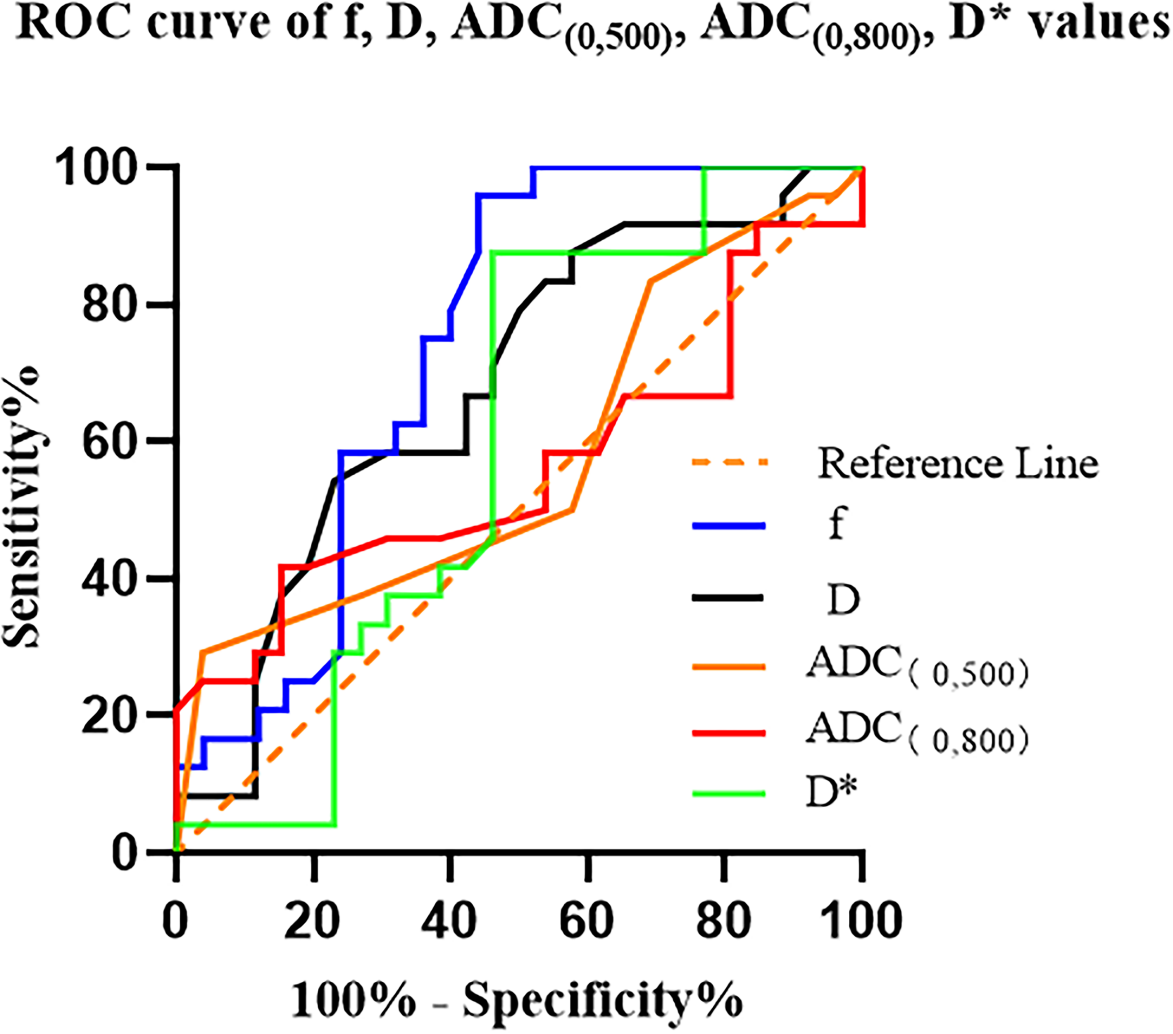
ROC curves for the ADC _(0, 500)_, ADC _(0, 800)_, D, D*, and f values for differentiating high- and low-fibrosis group PDAC D* is a specific parameter value of the IVIM, where * has no specific meaning..

To differentiate between high- and low-fibrosis group PDAC, the results of the ROC curve analysis for ADC _(0, 500)_, ADC _(0, 800)_, D, D*, and f values are listed in **Table 2**. The range of AUC is 0.561–0.742 (**Table 3** and **Figure 6**).

**Table 3:** ROC curve of ADC _(0, 500)_, ADC _(0, 800)_, D, D*, and f values.

	ADC _(0, 500)_	ADC _(0, 800)_	D	f	D*
AUC (95% CI)	0.445 (0.270–0.621)	0.484 (0.316–0.652)	0.678 (0.528–0.829)	0.742 (0.595–0.884)	0.662 (0.439–0.885)
P	0.582	0.870	0.03	0.004	0.104
Cutoff value	1.25 × 10^−3^	1.15 × 10^−3^	1.27 × 10^−3^	13.1%	160.5 × 10^−3^
Sensitivity	100%	100%	76.9%	95.8%	63.6%
Specificity	20.5%	33.3%	54.2%	53.8%	82.1%
NPV	20.5%	33.3%	64.5%	93.3%	82.1%
PPV	100%	90.9%	68.4%	65.7%	63.6%

*P < 0.05.

## Discussion

In this study, a significant correlation was established between the IVIM parameters and tumor fibrosis using the IVIM model. Compared to IVIM-DWI, ADC values have limited ability in grading fibrosis in PDAC. Intrareader and interreader agreement for ADC _(0, 500)_, ADC _(0, 800)_, D, and f values between different observers were excellent.

The ADC values are measured by DWI using a mono-exponential model, which reflects the diffusion motion of water molecules in tissues quantitatively. These ADC values could be influenced by cellular density, glandular formation, and fibrosis (21). Previous studies have shown that the ADC values of dense fibrosis PDAC were lower than those for the loose fibrosis group. However, in this study, we did not find any significant difference between ADC _(0, 500)_ or ADC _(0, 800)_ between high- and low-fibrosis PDAC groups. The differences in b values would affect the final ADC values (10–13, 21). When only low b-values are used, the signal is attenuated by the perfusion effect (22), while in high b-values, the diffusion effects cause a substantial signal attenuation (23). Standardization for DWI in the pancreas is still lacking. In the current Quantitative Imaging Biomarkers Alliance (QIBA) report, the liver is the only abdominal organ reported, with recommended maximum b values of 600 to 800 s/mm^2^ (22). None of these studies mention standardized pancreatic DWI or IVIM. Translating protocols from different organs to pancreatic imaging is challenging due to respiratory induced motion and different underlying physiology. Our study referred to previous studies in assessing the degree of fibrosis by DWI and found that the selection of maximum b values with 500/800 mm^2^/s was suitable in DWI (10, 11, 13, 23). Surprisingly, we received negative results with large cohorts. Curiously, we got negative results with bigger cohorts. Previous studies investigating multi-parametric models for IVIM in PDAC typically implement 3–10 b-values in the range of 0 to 1,000 s/mm^2^ (24). The downside of including a high b-value is the need for increased TE. The longer TE affects all acquired b-value images within the series, hence lowering the overall SNR and accuracy of the fitted model parameters. This is especially an issue in pancreatic tissue, which has short T2-times. As the non-diffusion parameters are less precise than the diffusion parameters, and as the non-diffusion effect occurs at low b-values, it is debatable whether multiparametric fits would desire a lower value for the highest b-value compared to the mono-exponential model. Hence we chose 10 b values and a maximum b value of 500 s/mm^2^ in our study. Some studies pointed out that the different choice of b values in DWI might not affect the diagnostic performance in breast lesions (22). In addition, the various scan parameters and fat-suppression technique might also affect the ADC value, and hence the absolute ADC threshold has a limited effect (22). Thus, a reduced field of view DWI was applied in the current study, and because some studies have pointed out that small vision DWI can reduce the artifacts and improve image resolution, the measured ADC value is rather accurate (25).

The signal attenuation on the DWI image consists of both true water molecular diffusion and random blood flow microcirculation perfusion, and the microcirculation perfusion superimposes the false diffusion signal on the diffusion image. IVIM distinguishes true water molecular diffusion from random blood flow microcirculation perfusion. The parameters of IVIM include D value representing true water molecule diffusion within voxels and f value representing the volume ratio of microcirculation perfusion effect within voxels to the total diffusion effect. The D values of the high fibrosis PDAC group were lower than those of the low fibrosis group in this study. Moreover, we found that the fibrosis was negatively correlated with the D values. Although all three factors, including fibrosis, glandular tissue, and tumor cells, affect the D values, fibrosis is the most dominant in PDAC. The effect of glandular tissue and tumor cells on D values is negligible compared to fibrosis (21); the higher the fibrosis component, the lower the D values.

F values represent the volume ratio of the microcirculation perfusion effect within voxels to the total diffusion effect, depending on the number and volume fraction of the capillaries (11). The f values of the high-fibrosis PDAC group were higher than those of the low-fibrosis group. In this study, we found that the f values were slightly positively correlated with fibrosis. The conversion of quiescent to activated pancreatic stellate cells (PSCs) drives the severe stromal reaction that characterizes PDAC (26). Furthermore, activated PSCs cause a severe stromal reaction, which is the feature of PDAC. Hypoxia induces profibrogenic and pro-angiogenic responses in PSCs (27). Therefore, we concluded that the microvascular is more abundant in high-fibrosis PDAC. As a result, both diffusion and perfusion effects are crucial in high-fibrosis PDAC (28).

The D* values mainly reflect the microcirculation perfusion of the tumor capillary network (29). The quantitative parameter D* values in this study were positively correlated with microvascular density. Previous studies demonstrated that perfusion-sensitive D* values could accurately detect intratumor vascular perfusion (30). Nonetheless, IVIM parameter reproducibility was moderate to excellent for f and D values, while it was less reproducible for D* (31). These results indicated that D* values could not be reliable quantitative parameters for perfusion analysis. Furthermore, since the D* values are mainly determined by the slope of the fast-decreasing part of the DW signal curve (typically <100 s/mm^2^ in the lower range of b values), an accurate estimation of the D* values requires multiple data sampling in the lower range, because low b values produce DWI images with high signal-to-noise ratios, which is often impractical. Also, D* values should be interpreted with caution. Finally, the tumor cell density was not associated with quantitative diffusion parameters using mono- or biexponential fitting, which is consistent with Xie et al. based on mono-exponential fit (32).

As a matter of fact, previous studies in assessing the grade of PDAC fibrosis were inconsistent. We found that there was no difference in the ADC values of high and low fibrosis PDAC, while the IVIM parameter values showed the better result than the ADC values. This study refers to part methods in Hecht’s research and innovates on its basis, especially in the selection of b values in DWI and IVIM-DWI. The correlation between IVIM-DWI parameter value and fibrosis degree was approximately the same as that of Hecht’s (11). Lemke et al. suggested that in case of limited acquisition time, the b values should be chosen in the given order, but at least 10 b values should be used for current clinical settings when applied to pulmonary lesions (33). Although the same number of b values were used both in our study and Hecht’s studies, the numerical number of b values were different. Wan et al. suggested that the number of b values < 50 s/mm^2^ should be at least 2, to assess the perfusion fraction more accurately (34). The b values in this study were consistent with Wan’s recommendations. Although the sample size increased in our study, a weak positive correlation between the f values and fibrosis was obtained in our study, which implied that the choice of the b values was controversial and further study was needed. Yong et al. pointed out that liver D* and f values showed poor reproducibility between 1.5 T and 3.0 T platforms (35). Comparing with 1.5T MR in Hecht’s study, the higher signal-to-noise ratio and image resolution can be obtained by using 3.0 T MR, which may explain the differences.

Nevertheless, the present study has several limitations. Firstly, we used a retrospective design and performed our study at a single center. Secondly, unresectable PDAC was not included in our study because we were unable to obtain tissue specimens of unresectable PDAC. Finally, the scanning time of IVIM was longer than for conventional MR sequences, and the MR images may be influenced by breath motion.

In conclusion, D and f values derived from the IVIM model had higher sensitivity and diagnostic performance in grading fibrosis of PDAC compared to the conventional DWI model. Thus, IVIM-DWI may have the potential as an imaging biomarker for predicting the fibrosis grade of PDAC.

## Data Availability Statement

The raw data supporting the conclusions of this article will be made available by the authors, without undue reservation.

## Ethics Statement

The studies involving human participants were reviewed and approved by the Third Affiliated Hospital of Soochow University, Institutional Ethics Committee. Written informed consent for participation was not required for this study in accordance with the national legislation and the institutional requirements. Written informed consent was obtained from the individual(s) for the publication of any potentially identifiable images or data included in this article.

## Author Contributions

QL and JGZ performed and designed experiments, analyzed and interpreted data, and wrote the manuscript. QL, JGZ, MJ, YZ, JLZ, BL, JC, WX, and TC performed the experiments. JGZ, JC, JLZ and WX contributed to discussions and reviewed and edited the manuscript. All authors contributed to the article and approved the submitted version.

## Funding

This study has received funding by National Natural Science Foundation project of China; contract grant number: 81901696, 81971572; Natural Science Foundation project of Jiangsu Province; contract grant number: H2018089.

## Conflict of Interest

Author JZ was employed by company Philips Healthcare.

The remaining authors declare that the research was conducted in the absence of any commercial or financial relationships that could be construed as a potential conflict of interest.
